# Saponins from *Panax notoginseng* ameliorate steroid resistance in lupus nephritis through regulating lymphocyte-derived exosomes in mice

**DOI:** 10.3389/fphar.2022.946392

**Published:** 2022-09-23

**Authors:** Jia Chen, Qingyun Zhou, Ying Lu

**Affiliations:** ^1^ Second Clinical Medical College, Zhejiang Chinese Medical University, Hangzhou, China; ^2^ Department of Nephrology, Hangzhou Linping Hospital of Traditional Chinese Medicine, Hangzhou, China; ^3^ Department of Nephrology, Zhejiang Academy of Traditional Chinese Medicine, Hangzhou, China

**Keywords:** exosomes, Panax notoginseng saponins, steroid resistance, GEC, lupus nephritis, mice

## Abstract

Lupus nephritis (LN) is the most common and severe type of organ damage and an important primary disease in end-stage renal failure in patients with systemic lupus erythematosus (SLE). Clinical guidelines recommend steroid treatment, but steroid resistance has become a major factor leading to treatment failure and affecting prognosis. Our previous study demonstrated that Saponins from *Panax Notoginseng* (Panax ginseng saponins, PNS) could reverse steroid resistance of lymphocytes by downregulating P-glycoprotein (P-gp) expression and provide renal protection in LN mice, but the mechanism by which lymphocytes transmit these related messages to renal lamina propria cells is not clear. Therefore, we further elucidated this mechanism through holistic experiments. In this study, low-dose methylprednisolone (0.8 mg/kg/day, MP) was used to induce a steroid-resistant lupus nephritis (SR-LN) mouse model in weeks one to four, and a therapeutic steroid dosage (MP 12 mg/kg/day) or a combined PNS (PNS 100 mg/kg/day) treatment was administered from week five to eight. Lymphocyte-derived exosomes (Lyme-Exos) were isolated from the spleens of mice and injected into untreated homozygous LN mice for 14 days *via* the tail vein. At the end of the experiment, the efficacy and mechanism of action of different groups of Lyme-Exos on LN mice were observed. The results revealed that exogenously injected Lyme-Exos were effectively taken up by the kidney and affected the progression of kidney disease. Steroid-resistant lymphocyte-derived exosomes intervented with PNS significantly downregulated the levels of silent information regulator-related enzyme 1 (Sirt1), multidrug resistance gene 1 (*MDR1*), and P-gp in the renal cortex and glomerular endothelial cells (GECs); reduced serum autoantibody [antinuclear antibody (ANA) and anti-double-stranded DNA (dsDNA)] levels and inflammatory markers (WBC, PCR, and PCT); improved renal function; and attenuated urinary microalbumin excretion. Additionally, renal histopathological damage (HE staining) and fibrosis (Masson staining) were improved, and immune complex (IgG) deposition and membrane attack complex (C5b-9) production were significantly reduced; the gene levels of inflammatory factors (*INF-γ, MCP-1, IL-8, IL-17, vWF, VCAM-1, IL-1β, IL-6, PTX3*) in the renal cortex were downregulated. Taken together, this study showed that PNS may alleviate steroid resistance in GEC by interfering with steroid-resistant Lyme-Exos to ameliorate LN progression, which will likely provide insights into developing a new LN treatment.

## Graphical Abstract

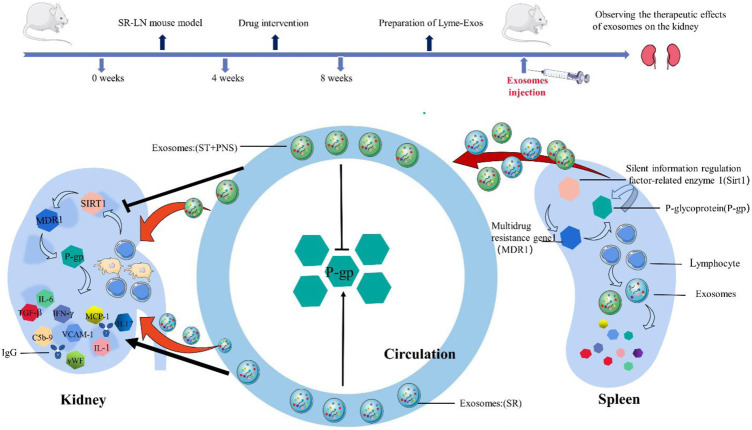

## 1 Introduction

Lupus nephritis (LN) is a serious complication of systemic lupus erythematosus (SLE). Approximately 50% of patients with SLE have LN as a complication. As medical care improves, the risk of developing end-stage renal disease remains unchanged, despite reduced mortality and improved disease prognosis in SLE patients (Almaani et al., 2017; Gasparotto et al., 2020). An efficient and low-toxicity treatment option is the key to the problem. Commonly used steroid drugs have anti-toxic, anti-inflammatory, and immune-suppressing effects. Regrettably, due to long-term treatment of LN, steroid resistance and concurrent infections are often induced, thus making treatment more challenging. The alternative use of other cytotoxic agents remains risky and challenging and is further complicated by the reduced efficacy of other immunosuppressive agents when steroid resistance occurs (Jobst-Schwan et al., 2019). Thus, developing an understanding of how steroid resistance can be reversed in LN is vital.

Recently, several studies have shown that drug resistance information can be transmitted by exosomes laterally through drug resistance-associated miRNAs, multidrug resistance proteins and soluble membrane receptors, thus resulting in the acquisition of the same resistance in distant tissue or cells (Batrakova and Kim, 2015; Kamerkar et al., 2017). Exosomes play a role in an intercellular communication mechanism that can regulate the gene expression profile of target cells over long distances while also playing an important role in the regulation of physiological functions, pathogenic injury, and organ remodeling (Théry et al., 2018; McAndrews and Kalluri, 2019).

As high-quality carriers of biological information and drugs, the value of exosomes has been recognized in multidisciplinary areas (Familtseva et al., 2019; Zhao et al., 2020; Chen et al., 2022). In kidney disease, exosomes can play a role in the mediation of intrinsic cellular crosstalk between glomerular and tubular cells to mediate organ crosstalk and participate in the amplification or inhibition of renal injury and inflammation (Grange and Bussolati, 2022). Furthermore, exosomes are released into the circulation from distant tissues and may contribute to intraorgan crosstalk and multiorgan dysfunctional conditions. Circulating exosomes from autoimmune diseases, such as SLE and antineutrophil cytoplasmic antibody (ANCA)-associated vasculitis, may be involved in renal pathology by promoting coagulation, thrombosis, and immune mediation (Mazzariol et al., 2021). Exosomes may similarly mediate the reversal of steroid resistance in LN, but little attention has been given to it.

As the first barrier to the glomerular filtration membrane, glomerular endothelial cells (GECs) are more susceptible to inflammation, proteins, lipids, and other biological molecules (Lassén and Daehn, 2020; Xu and Shao, 2022). One possible pathogenic mechanism responsible for LN involves SLE leading to the excessive activation of immune cells. In addition, immune complexes are created and deposited along the vessel wall, thus destroying endothelial cell autoantibodies. As a result, vascular inflammation is promoted, the endothelium is damaged, and function is disrupted (Barile-Fabris et al., 2014; Liu and Kaplan, 2018; Ryan et al., 2022). The glomerulus is structured as a network of tiny capillaries. When splenic lymphocyte-derived exosomes (Lyme-Exos) transmit messages over long distances to the kidney, it is likely that GECs are the key receivers of such messages.

In our previous study, we found that *Panax ginseng* saponin (PNS) reversed steroid resistance in the lymphocytes of steroid-resistant lupus nephritis (SR-LN) mice *via* the SIRT1/FoxO1/MDR1 signaling pathway (Pan et al., 2022). However, how the drug resistance or reversion information contained in lymphocytes is transmitted to the kidney and the mechanism responsible for the development of steroid resistance in kidney cells are unclear. Therefore, in this study, we observed the effect of treatment with Lyme-Exos on the level of resistance and lesions in the kidneys of LN mice from the perspective of overall animal experiments. Our findings are expected to provide insights into designing a new therapy for treating LN.

## 2 Materials and methods

### 2.1 Chemicals

Methylprednisolone (MP) tablets were obtained from Pfizer Italia S.r.l. (New York, United States). *Panax notoginseng* total saponin was provided by Guangxi Wuzhou Pharmaceutical (group) Co., Ltd. (Guangxi, China).

### 2.2 Animals

Female MRL/MpJ-Fas lpr (MRL-lpr) mice (16 weeks of age) were purchased from Shanghai SLAC Laboratory Animal Co., Ltd. (Shanghai, China). The mice were maintained under specific pathogen-free (SPF) conditions in the laboratory animal research center of the Zhejiang Institute of Traditional Chinese Medicine. All animal experiments were approved by the Chinese Institutional Animal Care and Use Committee (Approval number: 2019–045).

### 2.3 Animal experiments

In the first part of this study, we used our previous methods (Pan et al., 2022) to develop an SR-LN model. We did this by performing continuous gavage with a low dose of MP tablets (0.8 mg/kg/day) for 4 weeks. Then, we detected the expression of P-glycoprotein (P-gp) and its reaction substrate Rh-123 in splenic lymphocytes extracted from the mouse model. Increased expression of P-gp and reduced expression of Rh-123 in the model mice compared with the untreated mice with LN indicated that the steroid-resistant mouse model had been successfully constructed. Next, the SR-LN mice were randomly divided into three groups: a steroid-resistant group (SR) (0.8 mg/kg/day, MP), a therapeutic dose of steroid group (ST) (SR with 12 mg/kg/day, MP), and an ST with PNS group (ST + PNS) (ST with 100 mg/kg/day, PNS); an LN group as a control (equal volume, PBS) was treated for 4 weeks. MP was administered by gavage, while PNS was injected into the intraperitoneal cavity. All parallel groups were administered PBS-based alternative drug interventions in the same manner. Four groups of mice were sacrificed after 8 weeks of intervention. Four groups of Lyme-Exos were extracted, their protein concentration was determined, and the Lyme-Exos were then stored at −80°C. In the second part of the study, the Lyme-Exos from different groups were injected at a dosage of 20 µg/day for 14 days into untreated mice with LN *via* the tail vein, and the groups were named LN/Exo, SR/Exo, ST/Exo, and ST + PNS/Exo. All animal experiments were performed in accordance with the ethical requirements for laboratory animals.

### 2.4 Flow cytometry

Flow cytometry was used to detect the expression of P-gp and Rh-123 in the splenic lymphocytes of the mice in the SR-LN model group and the LN group to confirm the successful construction of the SR-LN model. Cell suspensions (1.0×10^6^/ml) were washed twice with prechilled PBS and then centrifuged (300×g, 4°C, 5 min). The cells were then incubated with an anti-P-gp antibody (Santa Cruz Biotechnology, Santa Cruz, CA, United States) (0.1 μg/ml) for 30 min at room temperature. The cells were washed twice with cell buffer, centrifuged (300×g, 4°C, 5 min) and incubated with FITC-coupled goat anti-rat monoclonal antibody (Beijing Jiamei Biotech, Beijing, China) for 30 min in the dark. The cells were then washed twice with cell buffer, resuspended to a volume of 500 µl and analyzed by flow cytometry (FACS FC500; Beckman Coulter, Brea, CA, United States). An isotype-labeled FITC-coupled unrelated antibody was used as a negative control. The P-gp and Rh 123 expression of GECs was detected using the same method following an additional digestion step.

### 2.5 Preparation of exosomes

Based on the physical properties and composition of exosomes, we extracted exosomes using the classical differential ultracentrifugation method (Théry et al., 2006; Lobb et al., 2015). The exosomes were isolated from Lyme-Exo suspensions by extraction as follows: centrifugation (4°C, 300 × g, 10 min) was used to transfer the supernatant to a new tube, centrifugation (4°C, 3,000 × g, 15 min) was used to transfer the supernatant to a new tube, centrifugation was performed at 4°C, 10,000 × g for 30 min, and centrifugation was performed with 0.22 µm filters (Millipore Billerica, MA, United States) to remove bacteria from the supernatant. Finally, the precipitate was collected by ultracentrifugation (4°C, 100,000×g, 2 h) as exosomes. Exosomes were identified by transmission electron microscopy (morphological structure) and nanoparticle tracking analysis (NTA) (particle size), and capillary immunoassays were used to detect characteristic marker proteins (TSG101 and Hsp70).

### 2.6 Capillary immunoassays

For the quantitation of proteins, we used a partially automated Simple Western system (WES) that was based on capillary electrophoresis; reagents were provided by the manufacturer (WES, Protein Simple, San Jose, CA, United States) (Nguyen et al., 2011). Exosomal proteins were extracted by adding an equal volume of exosome-specific lysis solution (Umibio, Shanghai, China) to the exosome suspension and lysing it on ice for 5 min. The lysates were then centrifuged (12,000×g, 4°C, 5 min) to obtain a supernatant that contained exosomal proteins. The processing of renal tissue and the extraction of total protein from GECs were performed as described previously. Protein concentrations were determined with a BCA Protein Assay Kit, and equal amounts of protein samples were used for capillary immunoblotting. In accordance with the manufacturer’s instructions, the levels of key proteins were determined by incubation with specific primary monoclonal antibodies against TSG101 (1:100; Abcam, Cambridge, MA, United States), Hsp70 (1:50; Abcam, Cambridge, MA, United States), Sirt1 (1:30; Abcam, Cambridge, MA, United States), P-gp and GAPDH (1:100; Abcam, Cambridge, MA, United States). Signals were detected by an HRP-conjugated secondary anti-rabbit antibody, and chemiluminescence from the samples was visualized using protein simple compass software. We calculated the area of immunopositivity for each target protein and divided this by the area of immunopositivity for the internal reference GAPDH.

### 2.7 Extraction, culture and identification of primary glomerular endothelial cells

Tissue samples of the kidney cortex were cut into small pieces (1 mm × 1 mm) and digested with 0.1% type IV collagenase (STEMCELL Technologies Inc., Vancouver, Canada) at 37°C for 30 min. The digested kidney cortex was sequentially passed through 80, 100, 200, and 400 mesh sieves and gently ground on the sieves to remove excess tubules and cellular debris. The 400 mesh sieves eventually left only glomeruli. The glomeruli were washed with endothelial cell complete medium, and the suspension was incubated at 37°C with 5% CO2, during which the GECs wrapped around the glomeruli and crawled outwards (Figure 7A). Formal identification was provided by staining for key endothelial cell markers (vWF and CD31; Abcam, Cambridge, MA, United States) and detecting endothelial cell marker proteins (CD31, vWF and VE-calmodulin; Abcam, Cambridge, MA, United States) using flow cytometry.

### 2.8 Urinary and blood analysis

The day before the end of the experiment, each mouse was placed in a different metabolic cage for urine collection. During urine collection, the mice were deprived of food but allowed to drink freely. The collected urine was centrifuged at 3,000 × g for 15 min at room temperature, and the supernatant was used to detect urinary microalbumin levels. Upon sacrifice, whole-blood samples were collected from the mice, and 150 μl was dispensed into anticoagulated vacuum tubes for blood analysis and testing. The remaining whole blood was placed in a pro-coagulation vacuum tube for 1 h at room temperature and centrifuged at 3,500 × g for 10 min at 4°C, and the serum was collected for the detection of blood creatinine and urea nitrogen. The clinical laboratory of Zhejiang University of Traditional Chinese Medicine was commissioned to test these key indexes.

### 2.9 Hematoxylin-eosin staining and masson’s staining

Renal cortical tissue was fixed in formalin for 24 h and then embedded in paraffin. Two sections (4 μm in thickness) were cut, dewaxed and dehydrated. One section was subjected to hematoxylin and eosin staining, dehydrated and sealed; the other section was stained with Masson’s stain in accordance with a Masson’s kit (HaoKe Biotechnology Co., Ltd., Guangzhou, China); sections were treated overnight with potassium dichromate, followed by Lixin red staining, phosphomolybdate treatment, aniline blue staining, differentiation, and dehydration prior to being sealed. Images were acquired and analyzed by microscopy (Nikon Instruments Inc., United States).

### 2.10 Immunohistopathological analysis

Two 4 µm kidney sections were mounted on slides and rehydrated followed by overnight incubation (separately) with rabbit anti-mouse P-gp (1:500, Abcam, Cambridge, MA, United States) and rabbit anti-mouse Sirt1 (1:500, Abcam, Cambridge, MA, United States). Antigen retrieval was conducted in heated 10 mM citrate buffer for 10 min. Slides were incubated with an HRP-labeled goat anti-rabbit general-purpose secondary antibody (1:200, Abcam, Cambridge, MA, United States) for 30 min. Subsequently, stained histological sections were observed at 400× magnification with a light microscope (Nikon Eclipse TE 2000-U microscope, Japan).

### 2.11 Immunofluorescence staining

First, we identified GEC staining. GECs in culture dishes were fixed in 4% paraformaldehyde after deculturing. Primary antibodies (vWF and CD31; 1:100; Abcam, Cambridge, MA, United States) were added and incubated for 1 h (4°C). Then, a goat anti-rat fluorescent secondary antibody IgG (1:500, Abcam, Cambridge, MA, United States) was added. Second, we stained GECs for the exosome uptake experiments. Exosomes were stained with PKH67 (1:100, Umibio, Shanghai, China) and cocultured with GECs for 24 h. The GEC cytoskeleton was stained with phalloidin (1:1,000, Abcam, Cambridge, MA, United States) and DAPI (BOSTER, Wuhan, China). Third, 4 μm kidney sections were dewaxed, rehydrated, and antigenically repaired. A range of monoclonal primary antibodies were then added and incubated overnight at 4°C for immunostaining. The primary antibodies were C5b-9 (1:300 Biss Antibodies, Beijing, China) and IgG (1:200, Abcam, Cambridge, MA, United States). The renal expression of the target antigens (IgG, C5b-9) was then determined, and fluorescent secondary antibodies for color development were added the next day. All fluorescence staining was performed in the absence of light following the addition of fluorescent secondary antibodies; a final drop of fluorescence quenching solution (Vector Laboratories, United States) was added to seal the slides. Images were acquired and analyzed using a fluorescence microscope (Nikon Eclipse TE 2000-U microscope, Japan).

### 2.12 ELISA quantification

Mouse serum samples were prepared as described previously. Samples were evaluated with ELISA kits. An ELISA kit for P-gp was obtained from ELK Biotechnology (Wuhan, China), and ELISA kits for ANA and dsDNA were obtained from Alpha Diagnostic Intl. Inc. (San Antonio, United States). ELISA kits for CRP and PCT were obtained from mlbio (Shanghai, China). These assays were performed using the reagents provided in the kits and in accordance with the manufacturer’s instructions.

### 2.13 Quantitative real-time polymerase chain reaction analysis

Experimental methods of Quantitative real-time polymerase chain reaction (qRT**‒**PCR) were performed as described previously (Luo et al., 2021) in accordance with the instructions provided by the manufacturer. First, total RNA was extracted from the renal cortex and GECs by TRIzol reagent. Then, RNA concentrations were determined by a nanodrop detector (MA, United States) and reverse transcribed into cDNA with a PrimeScript RT reagent kit (TaKaRa, Dalian, China). Then, RT‒PCR was performed on a 7500 StepOne Plus system (Applied Biosystems, Foster, CA, United States). Next, we used a TB Green Premix Ex Taq^TM^ II Kit (TaKaRa, Dalian, China) to detect the expression levels of *INF-γ*, *MCP-1*, *IL-8*, *IL-17, vWF*, *VCAM-1*, *IL-1β*, *IL-6*, *PTX3*, *MDR1, Sirt1, P-gp,* and *β-actin*. First, the samples were denatured at 95°C for 30 s (1 cycle). In the second stage, the samples were exposed to the following cycle: 95°C for 5 s and 60°C for 30 s (40 cycles). Extension was carried out at 95°C for 15 s, 60°C for 60 s, and 95°C for 15 s (1 cycle). The ten primers were obtained from Sangon Biotech (Shanghai, China). The primer sequences are provided in Table 1. Fold change was calculated by the 2^−△△Ct^ method. The endogenous reference genes for *INF-γ*, *MCP-1*, *IL-8*, *IL-17, vWF*, *VCAM-1*, *IL-1β*, *IL-6*, *PTX3*, *MDR1, Sirt1,* and *P-gp* was *β-actin*.

**TABLE 1:** Primer sequences of the target genes used in RT PCR analysis.


MDR-1	F-GCTGTCAGCCCTCTTATTGGAT
	R-GCTTTTGCATAAGCCTGGAGTTC
IFN-γ	F-CTGGAGGAACTGGCAAAAGGATGG
	R-GACGCTTATGTTGTTGCTGATGGC
MCP-1	F-CCACTCACCTGCTGCTACTCATTC
	R-CTTCTTTGGGACACCTGCTGCTG
VCAM-1	F-GAGGGTGGTGCTGTGACAATGAC
	R-GGGTGGCATTTCCTGAGAGAAGC
vWF	F-CATGTGCCAACAGCCAGTCTCC
	R-GAAGGTGACGATGTGCCGAGTG
IL-1β	F-TCGCAGCAGCACATCAACAAGAG
	R-AGGTCCACGGGAAAGACACAGG
IL-6	F-CTTCTTGGGACTGATGCTGGTGAC
	R-AGGTCTGTTGGGAGTGGTATCCTC
IL-8	F-TCGGGAGACCTCTAGACACTTTGC
	R-GCCTGTCAAGCTGACTTCACTGG
IL-17A	F-TGATGCTGTTGCTGCTGCTGAG
	R-CACATTCTGGAGGAAGTCCTTGGC
PTX3	F-AGTGGCTGAGACCTCGGATGAC
	R-CTCGGTGGGATGAAGTCCATTGTC
Sirt1	F-CCAGACCTCCCAGACCCTCAAG
	R-GTGACACAGAGACGGCTGGAAC
P-gp	F-TCCACCTGTAGCCTGAAGACTATCC
	R-AAGAGGTCGGCAATGCTGTCAAC
β-actin	F-TATGCTCTCCCTCACGCCATCC
	R-GTCACGCACGATTTCCCTCTCAG

Expression levels were determined by normalizing the Ct values to two control genes.

### 2.14 Statistical analysis

The measurement data are expressed as the mean ± standard deviation. One-way analysis of variance (ANOVA) was used to compare the differences among different groups. Comparisons between two groups were performed by the *t* test or rank-sum test. Statistical significance was defined at *p* ≤ 0.05. The tools used for data processing were SPSS 25 (SPSS Inc., Chicago, IL, United States) and GraphPad Prism 9 (GraphPad Software, La Jolla, CA, United States). Image analysis was carried out with ImageJ software.

## 3 Results

### 3.1 Lyme-exos were successfully prepared and carried the same drug resistance information as the mother cells

**FIGURE 1:**
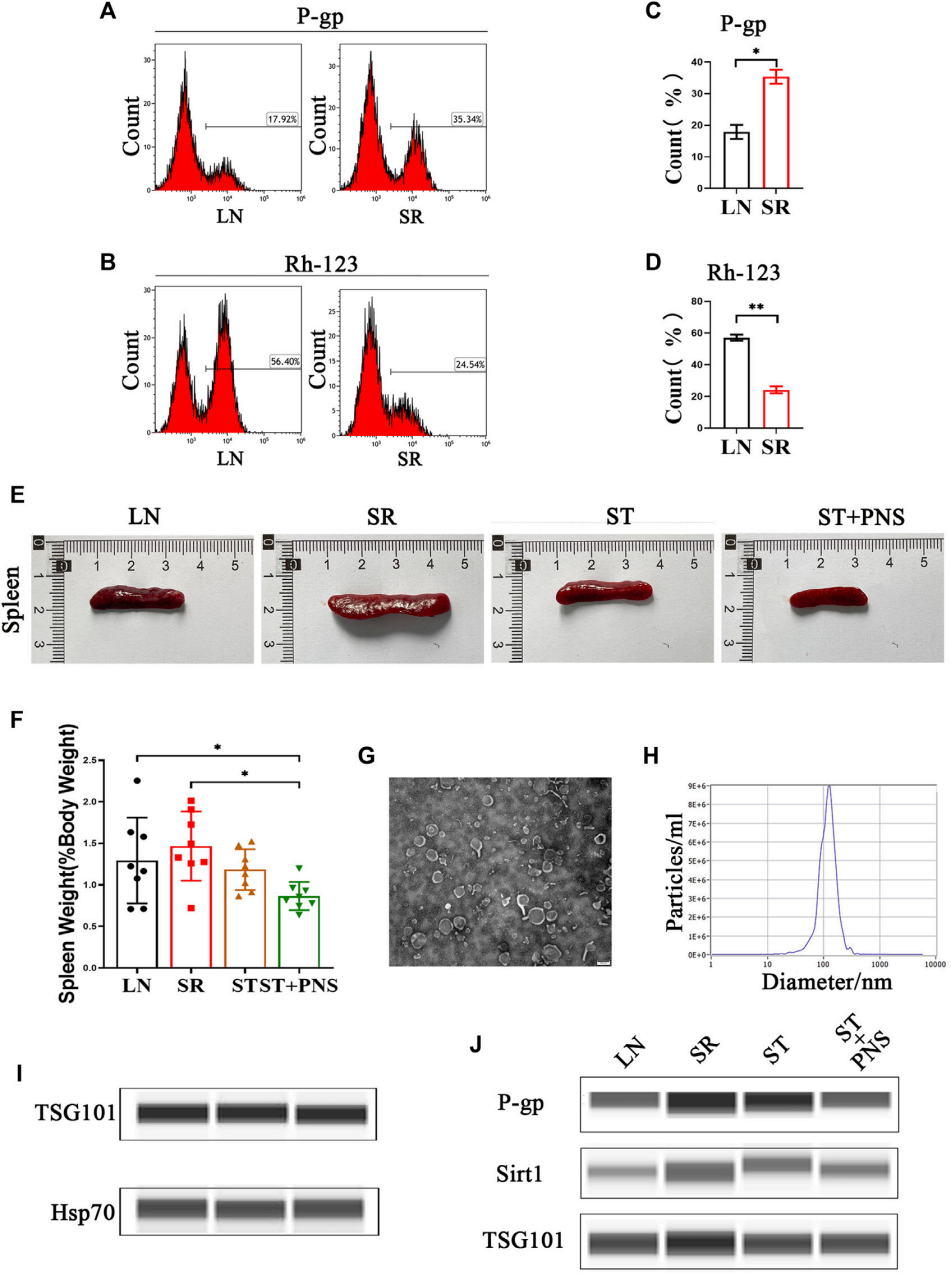
Validation of the SR-LN model and differential information expression of exosomes within the four groups. **(A–D)** Flow cytometry was used to detect P-gp expression and Rh-123 accumulation in splenic lymphocytes. **(E)** Images of one randomly selected mouse spleen per group and **(F)** The spleen index of each group of mice. **(G)** The morphology and structure of exosomes were photographed by transmission electron microscopy (scale bar 200 nm). **(H)** The particle size of the exosomes was detected by NTA. **(I)** Detection of the exosome markers TSG101 and Hsp70 by capillary immunoassays. **(J)** Capillary immunoassay analysis of exosome Sirt1 and P-gp protein expression in splenic Lyme-Exos extracted from each group. TSG101 is an exosome marker protein that acts as an internal reference. All data are expressed as the mean ± standard deviation from independent groups, *p < 0.05, **p < 0.01.

With 4 weeks of low-dose steroids, splenic lymphocytes of LN mice showed a significant increase in P-gp and a significant decrease in Rh-123 content, suggesting that the SR-LN model was established (Figures 1A–D). Based on this model, a 4-week intervention to reverse steroid resistance was performed in four groups: untreated mice with LN (LN), steroid-resistant mice with continuous low-dose steroid treatment (SR), mice with SR with added therapeutic doses of steroids (ST), and mice with ST with added PNS (ST + PNS). After treatment, there were differences in spleen size and splenic index between the four groups of mice, with a significant decrease in the ST + PNS group compared to the SR group (Figures 1E,F). The splenic Lyme-Exos were obtained from each group separately. Transmission electron microscopy revealed that the Lyme-Exos obtained by differential ultracentrifugation were high in quantity and low in impurities (Figure 1G). NTA experiments further confirmed that the proportion of exosomes that were 125.1 nm in diameter was 98.5% (Figure 1H). Capillary immunoassays confirmed that both TSG101 and Hsp70, characteristic markers of exosomes, were expressed in the extracted exosomes (Figure 1I). Analysis of exosomal capillary immunoassays for the four groups revealed that the protein levels of Sirt1 and P-gp were significantly higher in the SR group and significantly lower in the ST + PNS group (Figure 1J). The results showed that Lyme-Exos carried steroid resistance information consistent with the mother cells.

### 3.2 Kidneys can successfully take up circulating lyme-exos

**FIGURE 2:**
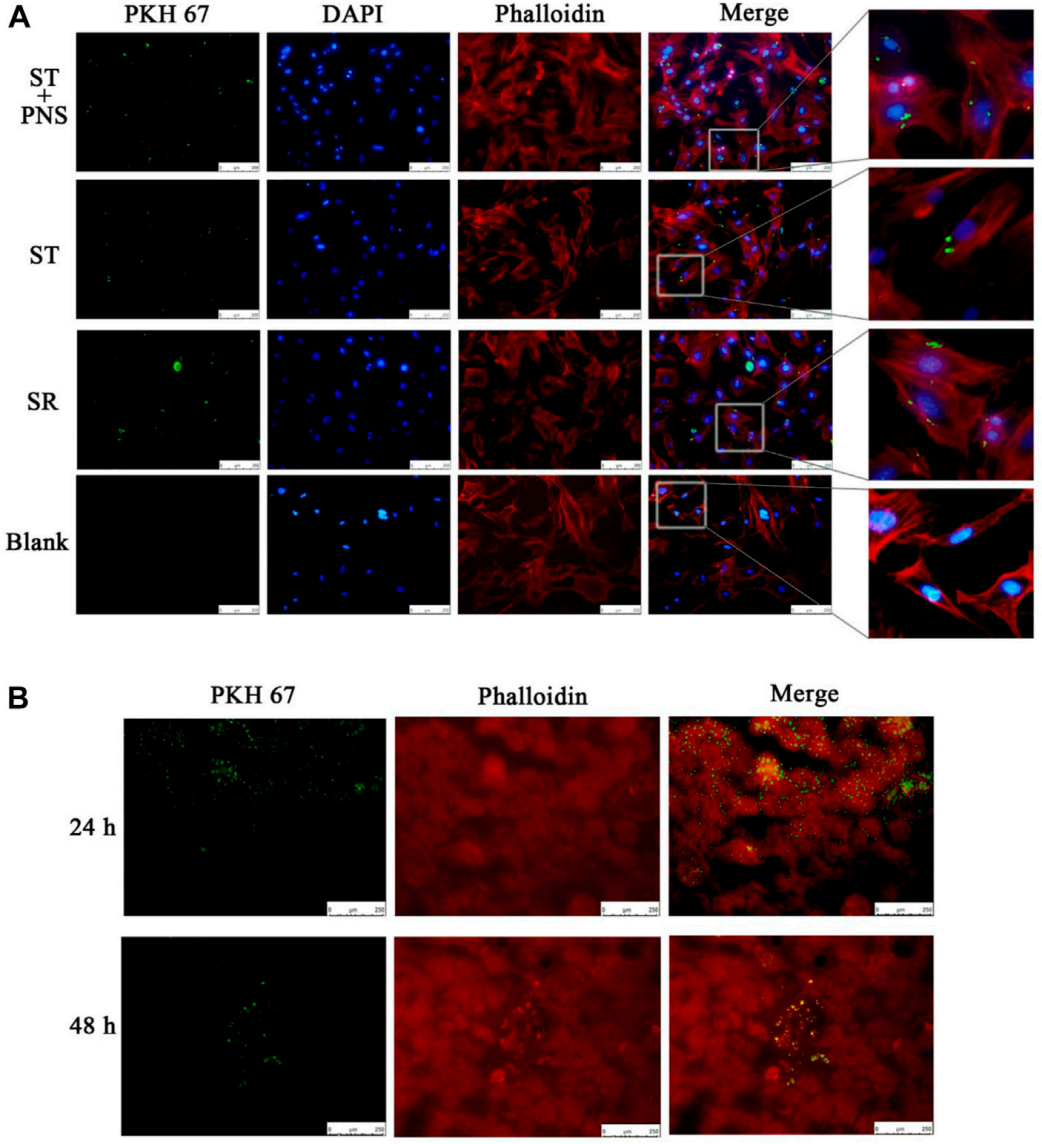
Levels of exosome uptake by kidney. **(A)** Uptake of PKH67-labeled exosomes (green fluorescence) by GECs (red and blue fluorescence, phalloidin and DIPA-stained cytoskeleton) in each group by cellular immunofluorescent staining. A blank control group was set up in parallel (scale bar 250 μm; enlarged image). **(B)** After the injection of PKH67-labeled exosomes into the tail vein, the mice were sacrificed at 24 and 48 h. The kidney cortex was taken for frozen sectioning. The uptake of PKH67-labeled exosomes (ultracentrifugation to remove excess dye) was observed in the kidney (scale bar 250 μm).

We cocultured each group of PKH67-labeled Lyme-Exos with primary GECs *in vitro* and found that GECs successfully internalized the exosomes (Figure 2A). In in vivo experiments, we injected stained Lyme-Exos into mice *via* the tail vein and observed that circulating exosomes were successfully enriched in renal tissue: they reached the kidney at 24 h and were enriched in the glomerulus at 48 h (Figure 2B). This finding indicates that the kidney can successfully take up circulating Lyme-Exos and that GECs may be one of the main cells involved in this process.

### 3.3 Panax ginseng saponins reverses the expression of renal steroid resistance messages by interfering with steroid-resistant lymphocyte-derived exosomes

#### 3.3.1 Overall results

**FIGURE 3:**
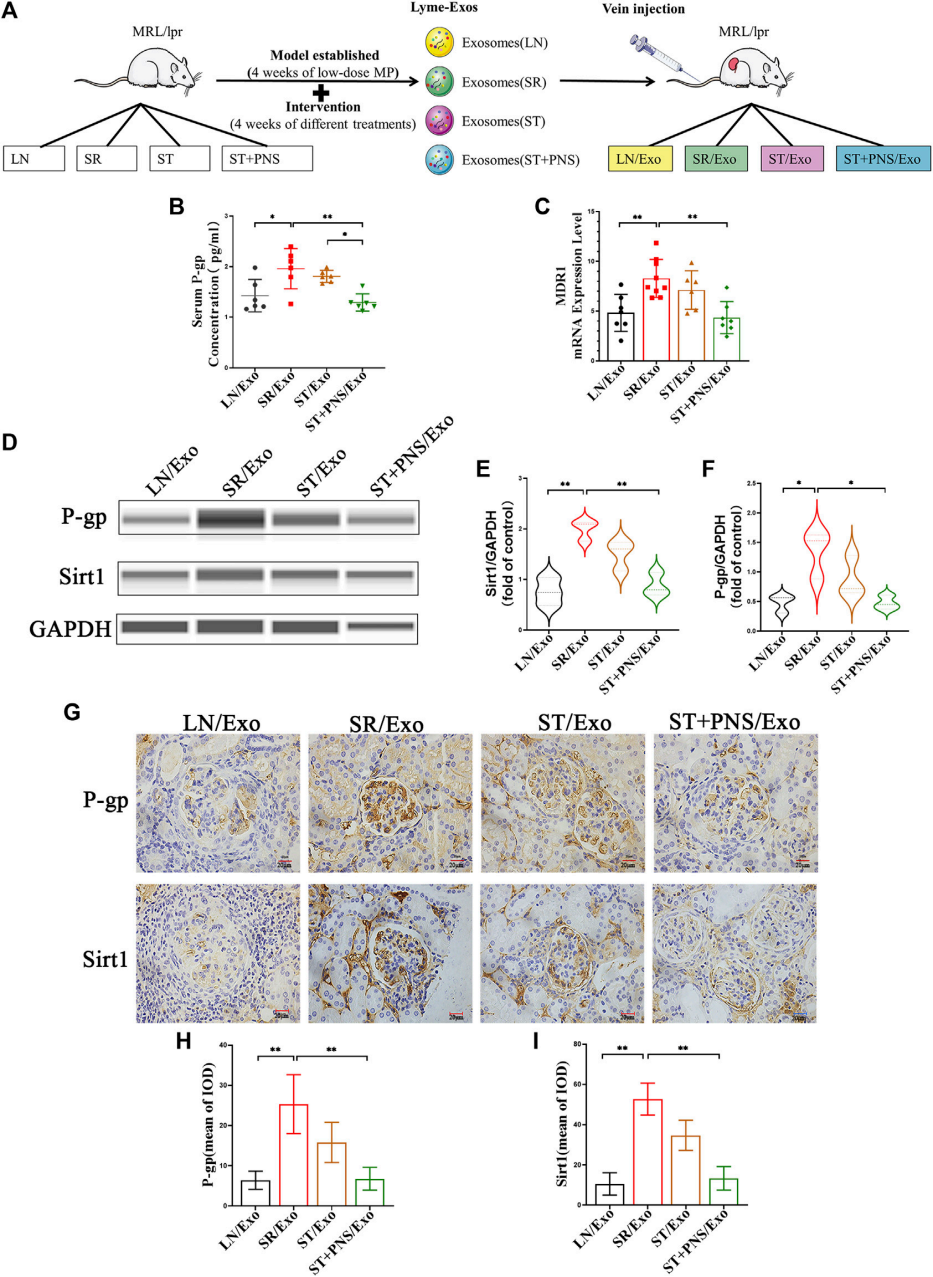
Information expression on drug resistance in serum and renal tissue after Lyme-Exo treatment. **(A)** Treatment flow chart and grouping information. **(B)** ELISA for serum P-gp levels. **(C)** Detection of *MDR1* gene expression of renal tissue by qRT-PCR. **(D–F)** Capillary immunoassay analysis of Sirt1 and P-gp protein levels of renal tissue. **(G–I)** Sirt1 and P-gp immunohistochemical staining (scale bar 20 μm). All data are expressed as the mean ± standard deviation from independent groups, *p < 0.05, **p < 0.01.

Untreated LN mice were randomly divided into four groups and were injected with exosomes obtained after 8 weeks of different drug interventions (the LN/Exo group was injected with exosomes from the LN group, the SR/Exo group was injected with exosomes from the SR group, the ST/Exo group was injected with exosomes from the ST group, and the ST + PNS/Exo group was injected with exosomes from the ST + PNS group) (Figure 3A). Compared with those in the SR/Exo group, the serum P-gp levels were significantly decreased in the ST + PNS/Exo group mice and were lower than those in the ST group (Figure 3B). Renal cortical *MDR1* mRNA (Figure 3C) and Sirt1 and P-gp protein expression were significantly downregulated (Figures 3D–F). Immunohistochemical results: the expression of glomerular Sirt1 and P-gp showed the same results (Figures 3G–I). It was suggested that PNS reversed the P-gp-mediated expression of the relevant steroid resistance message in the kidney by interfering with steroid-resistant Lyme-Exos.

#### 3.3.2 Cellular results

**FIGURE 4:**
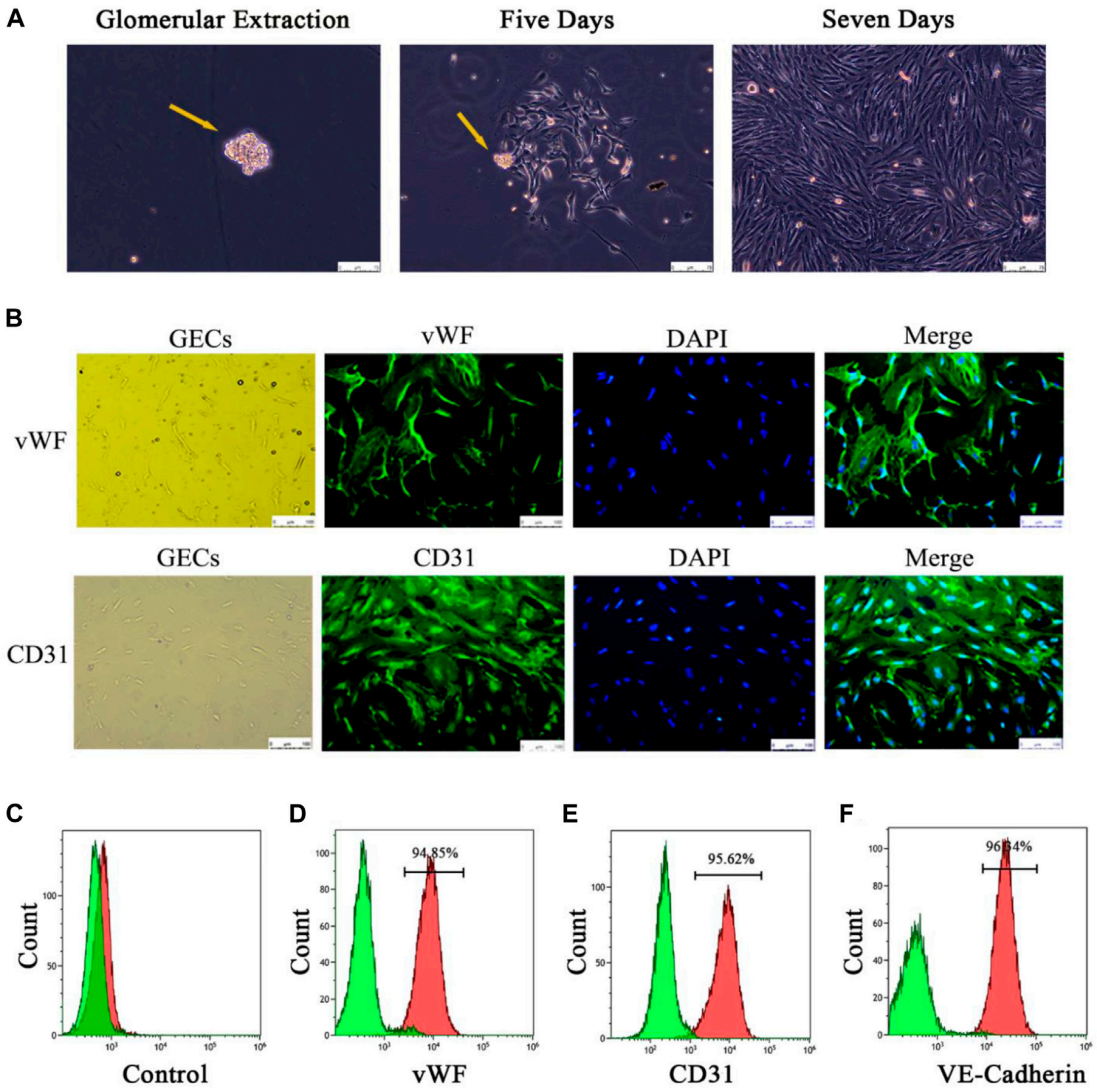
Extraction and identification of primary GECs from each group of mice after Lyme-Exo treatment. **(A)** Recorded the GECs crawling out of the glomerulus and dynamic growth process using a phase contrast microscope (yellow arrows represent glomeruli, scale bar 75 μm). **(B)** Immunofluorescence detection of the surface markers CD31 and vWF in GECs (scale bar, 250 μm). **(C)** Negative control group. **(D–F)** Flow cytometric detection of the marker proteins CD31, vWF, and VE-cadherin in GECs.

Four groups of mice GECs after different exosome interventions were extracted separately (Figure 4A) and identified. Immunofluorescence showed that almost all the cells expressed the endothelial cell surface markers CD31 and vWF (Figure 4B), and the expression rates of CD31, vWF and VE-cadherin in the extracted cells by flow cytometry were 95%, 94.85% and 96.34%, respectively (Figures 4D–F). It was confirmed that the extracted glomerular cells in each group were GECs.

**FIGURE 5:**
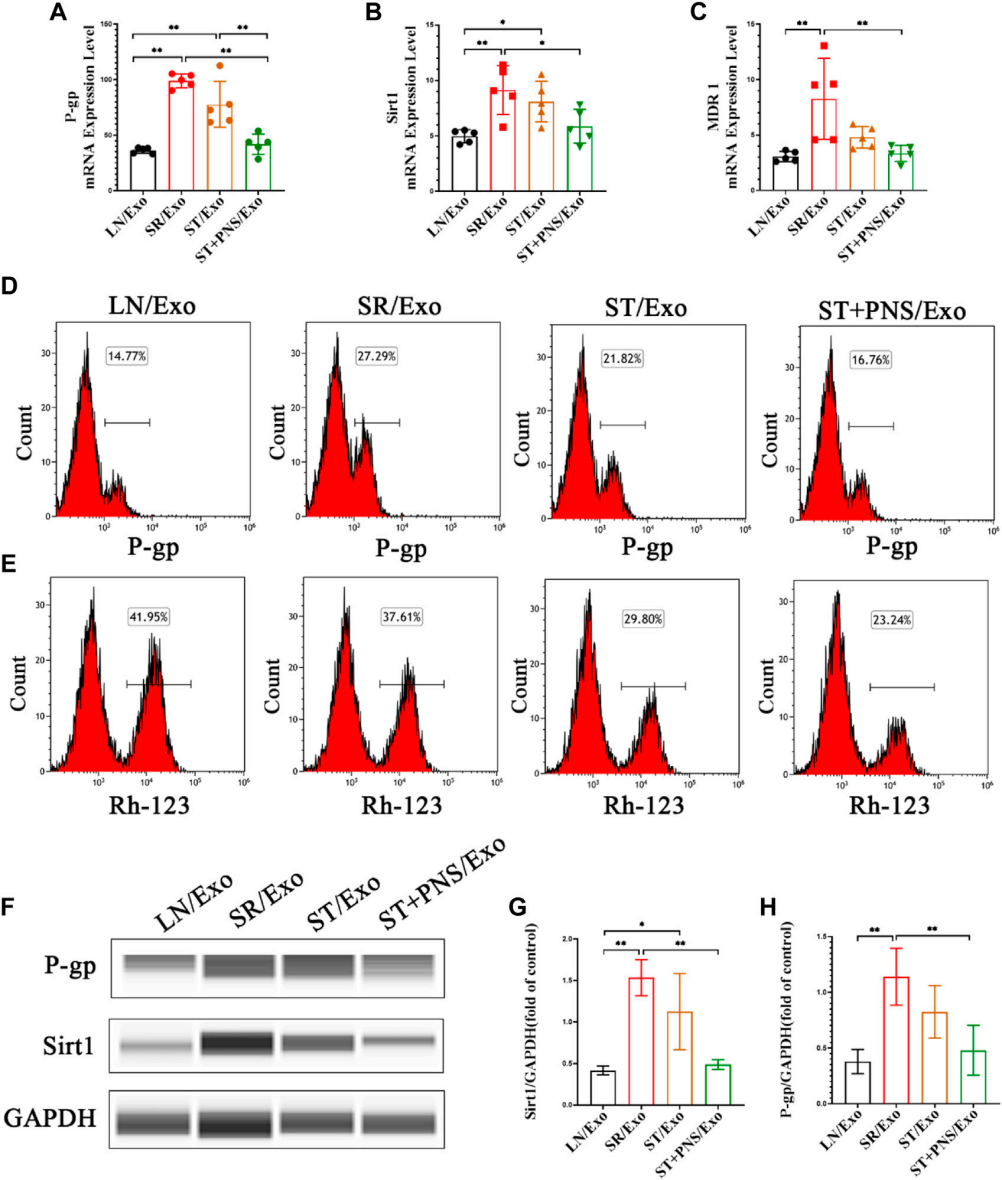
Expression of steroid resistance-related indicators in each group of GECs extracted after different Lyme-Exo interventions. **(A–C)** Detection of the mRNA expression of *Sirt1, MDR1* and *P-gp* by qRT-PCR. **(D,E)** Flow cytometry detection of P-gp levels and accumulation of Rh-123 in each group of GECs. **(F–H)** Sirt1 and P-gp protein levels in GECs were detected by capillary immunoassay. All data are expressed as the mean ± standard deviation from independent groups, *p < 0.05, **p < 0.01.

Detection of steroid resistance-related genes and proteins in the extracted primary GECs from each group revealed that GECs in the SR/Exo group expressed significantly higher levels of *P-gp, Sirt1*, and *MDR1* mRNA and Sirt1 and P-gp protein and that intracellular Rh-123 accumulation was significantly reduced (Figures 5A–H). In the ST/Exo group and ST + PNS/Exo group, the expression levels of *P-gp, Sirt1*, and *MDR1* mRNA (Figures 5A–C) and Sirt1 and P-gp protein (Figures 5D,F–H) in GECs were inhibited to different degrees, and the accumulation of intracellular Rh-123 was significantly higher, with the most pronounced effect in the ST + PNS/Exo group (Figure 5E). The results of this experiment showed that steroid-resistant lymphocytes delivered steroid resistance information to GECs through their secreted exosomes, and we similarly observed the effect of PNS on steroid resistance in GECs at the cellular level, suggesting that GECs, as one of the main effector cells, are involved in the regulation of steroid resistance in LN kidneys by Lyme-Exos.

### 3.4 Panax ginseng saponins reduced autoantibodies and inflammatory indexes and ameliorated renal injury in lupus nephritis mice by interfering with steroid-resistant lymphocyte-derived exosomes

#### 3.4.1 Improvement in serological parameters, renal function and renal pathological damage

**FIGURE 6:**
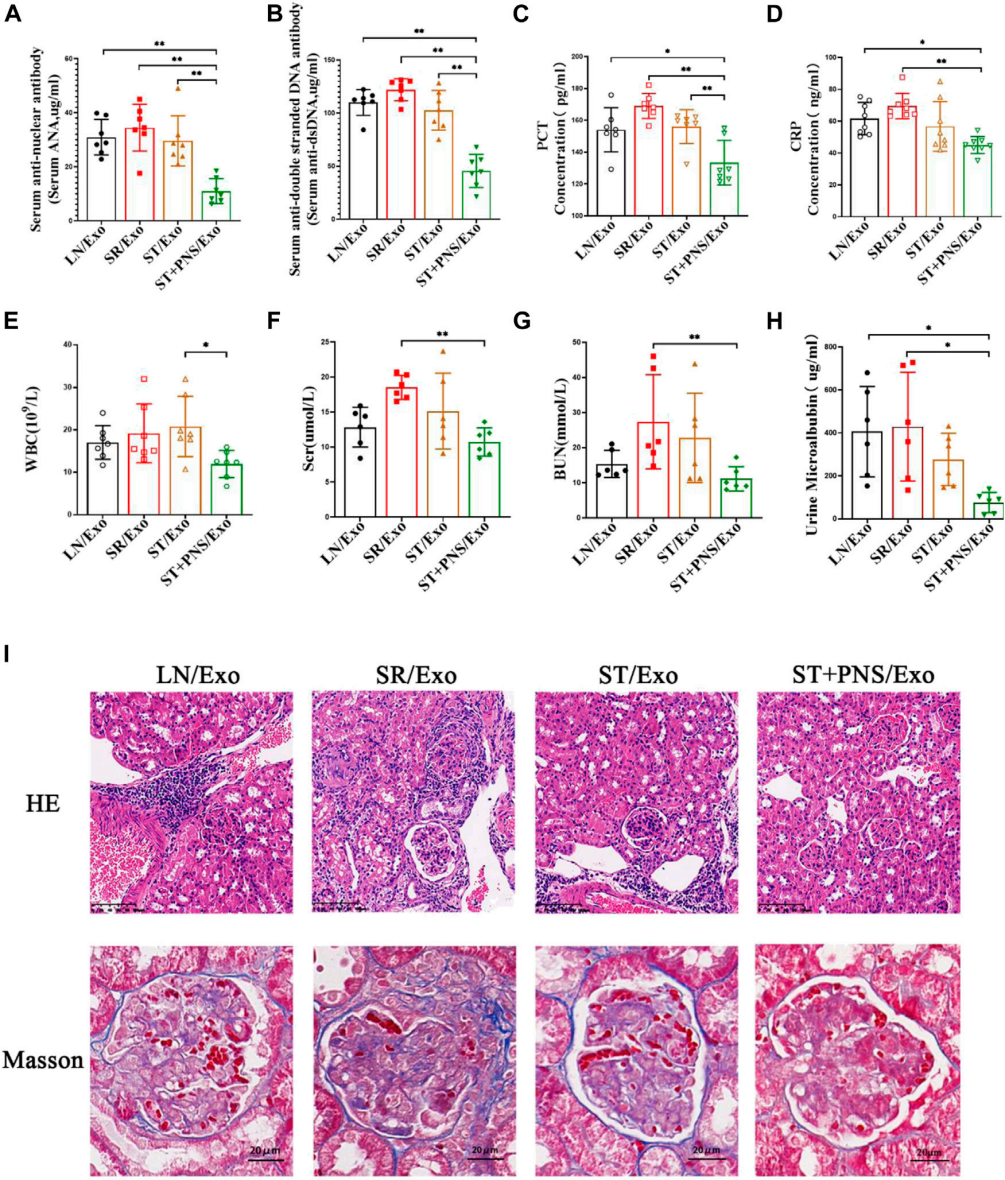
Evaluation of autoantibodies, inflammatory indexes, renal function, and renal pathological damage in each group of LN mice after different Lyme-Exo interventions. **(A–D)** Expression of serum autoantibody ANA, dsDNA, and inflammatory markers (CRP and PCT) by ELISA. **(E)** Whole-blood WBC assay by fully automated hematology analyzer. **(F–G)** Serum creatinine, urea nitrogen by automatic biochemical analyzer. **(H)** Urinary microalbumin excretion by fully automated protein analyzer. **(I)** Renal pathology HE and Masson staining (scale bars 100 μm and 20 μm). All data are expressed as the mean ± standard deviation from independent groups, *p < 0.05, **p < 0.01.

As shown in Figure 6, the mice in the LN/Exo and SR/Exo groups exhibited higher levels of serum autoantibodies and inflammatory markers, urinary protein, and serum creatinine and urea nitrogen. Meanwhile, obvious signs of renal injury such as inflammatory cell infiltration and glomerulophilic hemoglobin deposition in both groups. Serum autoantibodies (Figures 6A,B) and inflammatory markers (Figures 6C–E) were significantly decreased in the ST + PNS/Exo group. Serum creatinine and urea nitrogen of the ST + PNS/Exo group were not only lower than those in the LN/Exo and SR/Exo groups but also better than those in the ST/Exo group (Figures 6F,G). In addition, urinary microalbumin excretion was reduced (Figure 6H); renal tissue pathological damage was significantly improved in the ST + PNS/Exo group (Figure 6I).

#### 3.4.2 Diminished IgG and C5b-9 deposition in glomerular capillary collaterals

**FIGURE 7:**
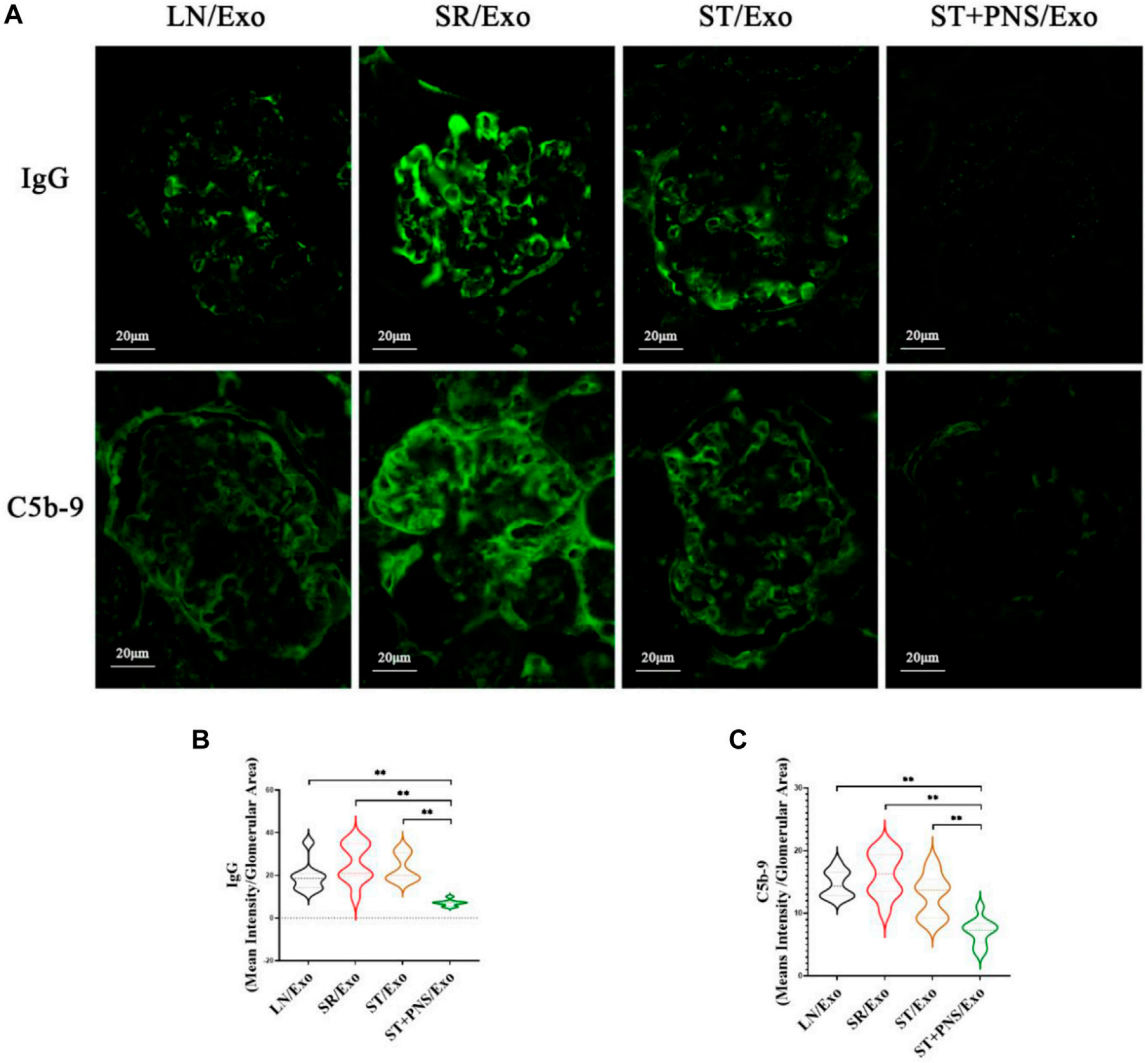
Effects of different exosome interventions on IgG and C5b-9 deposition in glomerular capillary collaterals of LN mice. **(A)** Glomerular IgG and C5b-9 expression in each group detected by Immunofluorescence staining of renal tissues (scale bar 20 μm). **(B,C)** Quantitative analysis of glomerular IgG and C5b-9 fluorescence intensity in each group (ImageJ software). All data are expressed as the mean ± standard deviation of independent groups, *p < 0.05, **p < 0.01.

Among the four groups, the glomerular immunofluorescence of mice in the SR/Exo group exhibited stronger immunoglobulin fine-granular deposition of IgG and membrane attack complex C5b-9 along the capillary collaterals (Figure 7A). In contrast, the deposition of IgG and C5b-9 in the glomerular capillary collaterals of LN mice in the ST + PNS/Exo group was significantly attenuated and even lower than that in the ST/Exo group (Figures 7B,C). This finding suggests that PNS/Exos contribute to the amelioration of glomerular immunopathological injury in LN mice.

#### 3.4.3 Downregulation of mRNA levels of multiple inflammatory factors in the renal cortex

**FIGURE 8:**
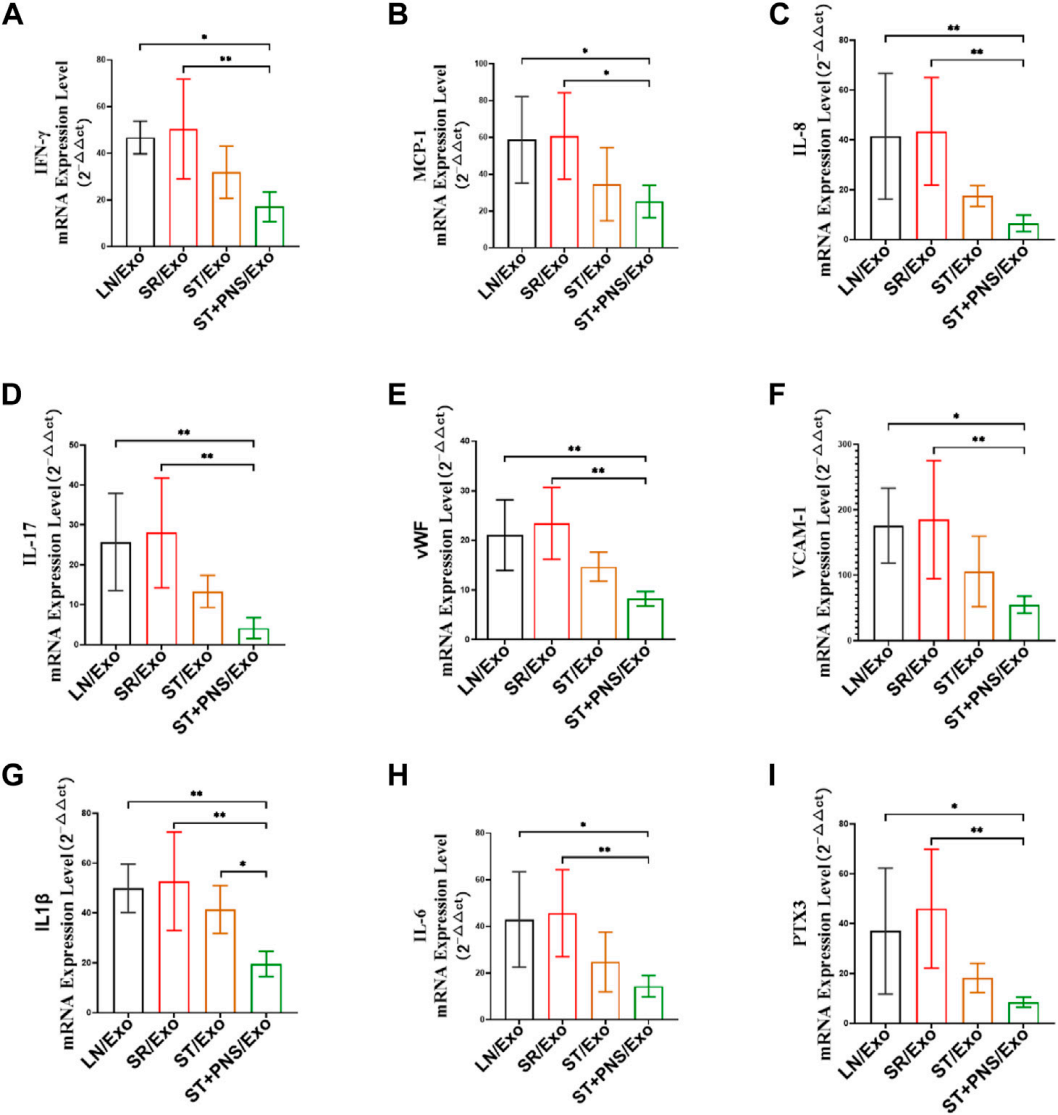
Renal cortical inflammatory factor mRNA expression was detected by qRT-PCR. **(A–I)** mRNA expression levels of *IFN-γ, MCP-1, IL-17, IL-8*, *vWF, VCAM-1, IL-1β, IL-6,* and *PTX3*. All data are expressed as the mean ± standard deviation from independent groups, *p < 0.05, **p < 0.01.

After different exosome interventions, we observed that the mRNA expression levels of various cytokines were regulated in the renal cortex. Such as IFN-γ, MCP-1, IL-8 and IL-17 secreted by activated T cells, (Figures 8A–D), adhesion molecules responding to vascular endothelial injury, such as VCAM-1 and vWF (Figures 8E,F), and various proinflammatory factors, such as IL-1β, IL-6, and PTX3 (Figures 8G–I) in ST/Exo and ST + PNS/Exo groups were decreased to different degrees, but only the ST + PNS/Exo group showed a statistically significant decrease.

## 4 Discussion

In this study, we investigated the effect of splenic Lyme-Exos on reversing steroid resistance in LN. The results of this study suggested that steroid-resistant lymphocytes deliver resistance information to the kidney by secreting exosomes that cross the circulatory system and that may also carry corresponding inflammatory factors and complement-activated components from the spleen (Figure 8). This phenomenon leads to the development of steroid resistance and the exacerbation of immune-inflammatory responses in the kidney, such as the marked increase in IgG in the kidneys of mice in the SR/Exo group. This immune complex further stimulates the complement system and the cascade reaction to form a membrane attack complex (C5b-9) that can trigger various inflammatory signals that subsequently promote inflammation and kidney injury (Ort et al., 2020) (Figure 7). In steroid-resistant splenic lymphocytes, the mechanism responsible for P-gp-mediated resistance in lymphocytes was damaged following PNS intervention (Figures 3, 4, 5). Exosomes secreted by effector lymphocytes carry key biological information for reversing drug resistance in the kidneys. The suppression of Sirt1 levels in the kidney downregulates the expression of the *MDR1* gene in renal tissues, thereby downregulating the levels of P-gp. Consequently, steroid resistance in the kidney can be reversed, thus enhancing sensitivity to steroid therapy drugs. This process also downregulates the inflammatory responses mediated by effector T cells in the kidneys, alleviates renal microvascular injury and improves the fibrosis caused by persistent inflammation (Figures 6, 8).

Drug resistance is a serious obstacle to the success of pharmacotherapy, and a common mechanism of drug resistance is the overexpression of ATP-binding cassette (ABC) efflux transporters (Amawi et al., 2019). P-gp is located in the cellular membrane and is a transmembrane ATP-dependent transporter molecule that responds to the efflux of drugs (Halder et al., 2022). The overexpression of P-gp and corresponding low expression of the P-gp substrate (Rh-123) is one main mechanism underlying the drug resistance of cells (Zhang Y. et al., 2016; Halder et al., 2022). Sirt1 is an NAD + -dependent deacetylase from the Sir2 family that regulates cell survival and longevity in many different organisms (Ceballos et al., 2018; Zheng et al., 2019). Overexpression of Sirt1 decreased Rh-123 staining and successfully increased the expression of MDR1/P-gp in R-HepG2 cells (Jin et al., 2015). Therefore, the search for Sirt1/P-gp inhibition strategies appear to be crucial for drug resistance. This study showed consistent results: PNS intervention with splenic Lyme-Exos is able to overcome drug resistance in LN-related cells, and the mechanism is related to the Sirt1/P-gp signaling pathway. The reduction in steroid outflow was reduced, thereby improving efficacy, which provides us with a new target for PNS treatment of steroid resistance.

PNS, a type of Chinese medicine that can activate blood stasis, has various pharmacological effects, including blood circulatory effects, antioxidative stress and blood pressure amelioration; immunity and blood sugar regulation; and anti-inflammatory action (Xi et al., 2000; Karu et al., 2007; Li et al., 2016; Zhang G. et al., 2016). The traditional Chinese medicinal pathology of LN is characterized by a deficiency of kidney and heart toxicity, with blood stasis running throughout the course of the disease. Successive clinical studies have also shown that the method of activating blood and resolving blood stasis can improve the clinical efficacy of LN (Pan et al., 2022). In this study, we performed an initial exploration of the mechanism underlying this important function. As an intermediate, exosomes can participate in proximal and distant intercellular communication and affect all aspects of cell biology (Pegtel and Gould, 2019). The functionality of exosomes is determined by the cell from which they originate and varies with the metabolic state and cellular environment of the cell (Skotland et al., 2017; Gao et al., 2018). In this experiment, we used the spleen as the main immune organ. The experimental results showed that the expression of drug resistance-related information in exosomes derived from splenic lymphocytes underwent changes when the mode of intervention was altered. In addition, we hypothesized that the mechanism by which exosomes achieve functional effects is as follows. The key active ingredient of PNS in the exosomes within lymphocytes and other small molecules can undergo complex interaction reactions and immune regulation in the drug-resistant environment of SLE splenic lymphocytes. When a certain time point is reached, exosomes are secreted by lymphocytes to target the kidney to exert biological effects. This speculation is consistent with the results of many studies (Kalluri and LeBleu, 2020). In the future, we will further identify the components of Lyme-Exos involved in this phenomenon.

The basic pathological manifestation of LN is well known to be microvascular inflammation. LN associated with vascular injury is predicted to result in severe renal damage and is considered an independent risk factor for poor renal prognosis (Tan et al., 2014). The pathological mechanism underlying LN microangiopathy is complex; studies have shown that the key pathological mechanisms of this disease are the activation of GECs, injury and the abnormal function of the immune system (Rodríguez-Carrio et al., 2012; Atehortúa et al., 2017; Atehortúa et al., 2019). In the present study, we used *in vitro* cell experiments to show that GECs from mice with LN can take up exosomes. The expression of Sirt1/P-gp in primary GECs extracted at the end of the experiment demonstrated that steroid resistance in the PNS group was also effectively reversed. Furthermore, mRNA detection of the renal cortex revealed the significant regulation of the endothelial-associated inflammatory factors VCAM-1 and vWF. Our conclusions indicated that splenic Lyme-Exos interact with endothelial cells and participate in the inflammatory responses of glomerular vascular endothelial cells and that the major intrinsic cells of exosome action in LN are likely to be GECs.

In general, this study indicated that exosomes of splenic lymphocytes under PNS interference are potential targets for reversing steroid resistance and can improve LN. The major strength of this study is that the activity and quantity of exosomes prepared from the mouse model using drug interventions *in vivo* are better than those obtained from *in vitro* coculture. Of course, this study has limitations, such as some heterogeneity among the LN mice. If artificial intelligence and other technological means can be used to load the active ingredients into exosomes in an efficient and standardized manner, then these exosomes may help in developing a new strategy for treating LN, and this will be the direction and goal of our future efforts.

## 5 Conclusion

Our study shows that steroid-resistant lymphocytes transmit steroid resistance information to GECs through their secreted exosomes, which exacerbates inflammatory lesions and histopathological damage in the kidney. PNS reverses steroid resistance in GECs by interfering with steroid-resistant Lyme-Exos, which results in varying degrees of inhibition of the secretion of various cytokines, proinflammatory factors, and vascular adhesion molecules. PNS/Exo also reduces the level of autoantibodies in the serum of LN mice, decreases urinary protein excretion, improves renal pathology, and thus protects renal function. Notably, the effect of ST + PNS/Exo is better than that of ST/Exo, suggesting that PNS can play a better role in the treatment of refractory LN in synergy with steroids. In addition, this study suggests that exosomes can be used as a medium to comprehensively explore the pathological mechanism of steroid resistance in LN and can be used as a carrier of therapeutic drugs to further investigate and develop the efficacy of traditional Chinese medicine treatments for LN.

## Data Availability

The datasets presented in this study can be found in online repositories. The names of the repository/repositories and accession number(s) can be found in the article/supplementary material.
